# STC1 and PTHrP Modify Carbohydrate and Lipid Metabolism in Liver of a Teleost Fish

**DOI:** 10.1038/s41598-018-36821-2

**Published:** 2019-01-24

**Authors:** Pedro F. S. Palma, Christian Bock, Tomé S. Silva, Pedro M. Guerreiro, Deborah M. Power, Hans-Otto Pörtner, Adelino V. M. Canário

**Affiliations:** 1 CCMAR/CIMAR – Centre of Marine Sciences, University of Algarve, Campus de Gambelas, 8005-139 Faro, Portugal; 2 AWI – Integrative Ecophysiology, Alfred-Wegener-Institute Helmholtz Centre for Polar and Marine Research, Am Handelshafen 12, 27570 Bremerhaven, Germany; 3 SPAROS, Lda., Área Empresarial de Marim, Lote C, 8700-221 Olhão, Portugal

## Abstract

Stanniocalcin 1 (STC1) and parathyroid hormone-related protein (PTHrP) are calciotropic hormones in vertebrates. Here, a recently hypothesized metabolic role for these hormones is tested on European sea bass treated with: (i) teleost PTHrP(1–34), (ii) PTHrP(1–34) and anti-STC1 serum (pro-PTHrP groups), (iii) a PTHrP antagonist PTHrP(7–34) or (iv) PTHrP(7–34) and STC1 (pro-STC1 groups). Livers were analysed using untargeted metabolic profiling based on proton nuclear magnetic resonance (^1^H-NMR) spectroscopy. Concentrations of branched-chain amino acid (BCAA), alanine, glutamine and glutamate increased in pro-STC1 groups suggesting their mobilization from the muscle to the liver for degradation and gluconeogenesis from alanine and glutamine. In addition, only STC1 treatment decreased the concentrations of succinate, fumarate and acetate, indicating slowing of the citric acid cycle. In the pro-PTHrP groups the concentrations of glucose, erythritol and lactate decreased, indicative of gluconeogenesis from lactate. Taurine, trimethylamine, trimethylamine N-oxide and carnitine changed in opposite directions in the pro-STC1 versus the pro-PTHrP groups, suggesting opposite effects, with STC1 stimulating lipogenesis and PTHrP activating lipolysis/β-oxidation of fatty acids. These findings suggest a role for STC1 and PTHrP related to strategic energy mechanisms that involve the production of glucose and safeguard of liver glycogen reserves for stressful situations.

## Introduction

In tetrapods, calcitonin and parathyroid hormone (PTH) act antagonistically to balance extracellular calcium. In teleost fishes, calcium balance is maintained by stanniocalcin 1 (STC1) and PTH-related protein (PTHrP) that have hypocalcemic and hypercalcemic actions, respectively^1–3^.

STC1 was first discovered in the corpuscles of Stannius (CS) of the sockeye salmon, *Oncorhynchus nerka*^4^, and its presence together with the isoform STC2, the product of a different gene, has now been established in all vertebrates^3,5–7^. Duplicate genes for STC1 and STC2 have been identified in teleost fishes but the isoform STC1A is the most abundant in the CS^7^. STC1A inhibits calcium uptake via the gills and intestine and stimulates phosphate reabsorption in the kidneys in order to maintain normal physiological levels of serum calcium and phosphate^3,8–10^. In mammals, STC1 and STC2 have a widespread tissue distribution and act as paracrine and autocrine factors with pleiotropic functions that include: calcium/phosphate transport in kidney and intestine^5,11,12^, bone and muscle structure and function^13^, anti-apoptotic effects on multipotent stromal cells^14^, protection against cellular hypoxia and hypertonic stress^15,16^, anti-inflammatory renal protective^17^, angiogenesis^18^, carcinogenesis^19^ and stimulation of the electron-transport chain in mitochondria^20^.

Despite growing interest in STCs, the receptors have not yet been isolated. However, STC binding is widespread in tissue, including mitochondria membranes of hepatocytes, myocytes and nephron cells, which together with the reported electron-transport chain stimulation suggests a role in cellular energy metabolism^20,21^. This is supported by recent *in vitro* studies which demonstrated STC1 modulation of renal gluconeogenesis in rat and fish^22^ and lipogenesis in rat white retroperitoneal adipose tissue^23^.

PTH and PTHrP share high N-terminal (1–34) sequence homology and bind with similar affinities to the same PTH receptor type 1 (PTH1R)^2,24^. In higher vertebrates, PTHrP acts as an endocrine, paracrine and autocrine factor, and has an important physiological role in growth and differentiation of bones, teeth and mammary gland development and the cardiovascular system^24,25^. Consistent with its pleiotropic roles the PTH1R has a widespread tissue distribution which includes liver^26–28^. As with STC, in teleost fishes PTH and PTHrP genes and receptors have undergone specific duplications^2^. The isoform PTHrPA circulates at high concentrations in blood plasma compared to the levels detected in human blood^29^ and is the only isoform with demonstrated calciotropic actions, increasing calcium uptake by the gills, kidney and intestine and reducing bicarbonate secretion^1,8,30–33^. PTHrPA has a widespread tissue distribution suggesting it has additional functions that remain to be deciphered^2,34^. To our knowledge, a single study links PTHrP to the composition of phospholipids in membranes^35^ while a few reports associate PTH with liver and renal gluconeogenesis, from alanine and lactate, respectively^36,37^, and lipolysis in adipose tissue^38,39^.

Here we tested the hypothesis, that STC1 and PTHrP have a role, possibly antagonistic, in intermediate metabolism. European sea bass (*Dicentrarchus labrax*) were treated with PTHrP(1–34) or its antagonist PTHrP(7–34), or a combination of PTHrP(1–34) and Anti-STC1A serum or STC1A and PTHrP(7–34). Liver metabolites of the treated and control groups were quantified using ^1^H-NMR at 6 h and 24 h. The results indicate significant changes in amino acid, carbohydrate and lipid metabolism, lending support to our hypothesis.

## Material and Methods

### Chemicals, Peptides and Proteins

Chemicals used to prepare solutions and buffers were of high-quality reagent grade from Sigma-Aldrich (Madrid, Spain), unless stated otherwise. PTHrP(1–34) and PTHrP(7–34) were synthetized by Genemed Synthesis, Inc (San Francisco, CA) and were based on the puffer fish, *Takifugu rubripes*, PTHrPA sequence, which is highly conserved across vertebrates^1^. STC1A was extracted from European sea bass CS and purified by SDS-PAGE using a Prep Cell Model 491 (Bio-Rad)^8^. The anti-sea bream (*Sparus aurata*) STC1A serum was the one from Gregorio *et al*.^40^.

### Experimental animals

European sea bass juveniles of 15.94 ± 0.83 cm fork length and 43.8 ± 7.2 g were obtained from a local fish farm (AquaAlvor, Lda) and transported to the Ramalhete Marine Station at the University of Algarve (Faro, Portugal). Fish were maintained in 500 L flow-through seawater tanks (37 ppt salinity) under natural temperature (23 °C) and photoperiod and fed with commercial dry pellets at a rate of 1% body weight/day. Animal experimentation was conducted in May 2014 in accordance with the Guidelines of the European Union Council (Directive 2010/63/EU) transposed to national Decree-Law 113/2013. In the absence of an animal welfare committee at the time (the legislation was only introduced in January 2015), experiments were carried out under a “Group-1” licence issued by the Ministry of Agriculture, Rural Development and Fisheries of Portugal.

### Hormone treatments

A month before the experiments, the fishes were randomly distributed between 500 L tanks and left to acclimatize. Fishes were fasted for 24 h prior to experimental manipulations. Fish were anesthetized in 2-phenoxyethanol (1:10000) and were given intraperitoneal injections of the following treatments (based on^40^): 1) 0.9% of NaCl vehicle solution (5 μL/g; control); 2) 0.5 μg/g PTHrP(1–34); 3) 0.5 μg/g PTHrP(1–34) plus 1 μL/g anti-STC1A rabbit serum; 4) 0.5 μg/g PTHrP(7–34); 5) 0.5 μg/g PTHrP(7–34) with 2.5 μg/g STC1A. Assuming PTHrP and STC1 are antagonistic, the treatment with PTHrP(1–34) plus anti-STC1A rabbit serum should amplify the effect of PTHrP(1–34), while the treatment of PTHrP(7–34) plus STC1A should amplify the effect of STC1A through inhibition of PTHrP. Hereafter, group 2) and 3) will be referred to as pro-PTHrP groups and 4) and 5) as pro-STC1 groups. After injections, fishes were transferred back to their tanks and were euthanized at 6 h and 24 h post-injection, (n = 10 per group), with an overdose of 2-phenoxyethanol (1:250).

Fish were measured and weighed, and blood collected in heparinized syringes (500 U/mL ammonium heparin). Plasma was separated by centrifugation and stored at −80 °C for analysis. Tissues were immediately dissected, frozen in liquid nitrogen and stored at −80 °C.

### Blood plasma biochemistry

Plasma free and total calcium, phosphorus, glucose and lactate levels were quantified using commercial colorimetric assays (respectively Spinreact refs 1001061, 1001155, 1001190 and 1001330, Barcelona, Spain). Free calcium was measured by filtering the plasma samples through a centrifugal filter with polyethersulfone (PES) membrane with 10 K molecular weight cut-off (VWR international, Lisbon, Portugal). Total plasma protein was quantified in duplicate using the Bio-Rad Protein Assay Dye Reagent Concentrate (ref 500-0006) with bovine serum albumin (BSA) as standard (ref 500-0207 in a Benchmark Microplate Reader (Bio-Rad, Hercules, CA, USA). Plasma osmolality was determined using a VAPRO® Vapor Pressure Osmometer, model 5520 (Wescor, Inc., USA) calibrated every 20 measurements.

### Extraction and identification of metabolites from liver samples

Metabolite extraction from liver samples followed previously reported methodology^41^. Briefly, frozen samples (~0.3 g per sample) were powdered with a mortar and pestle under liquid nitrogen and extracted in 0.6 M perchloric acid. Extracts were frozen at −80 °C in glass vials, lyophilized, stored in a nitrogen atmosphere and shipped to the Alfred-Wegener-Institute for metabolite identification and quantification using untargeted metabolic profiling based on ^1^H-NMR spectroscopy as described recently in^42^. Briefly, samples were resuspended in D_2_O containing 1% trimethylsilyl propionate (1:1 g/mL, TSP). The extract (70 μL) was transferred into a standard zirconium rotor for high-resolution magic angle spinning (HRMAS) ^1^H-NMR spectroscopy. NMR spectra were recorded at room temperature in a 400 MHz 9.4 T NMR spectrometer (400 WB Avance III, Bruker Biospin, Germany) at a spinning rate of 3000 Hz. Four typical NMR sequences for metabolite profiling were recorded for each sample as described in^42,43^: a. 1D onepulse sequence with f1 pre-saturation, b. 1D Carr-Purcell-Meiboom-Gill (CPMG) sequence, c. NOESY sequence and d. a pseudo 2D J-resolved (JRES) protocol. Spectra were acquired with 32 scans. The data of the CPMG sequence were used for metabolite identification and quantification using Chenomx NMR suite 8.0 (Chenomx Inc., Canada). A line broadening of 5 Hz was applied before fourier transformation. All spectra were calibrated and shim corrected on the internal TSP standard, followed by operator-controlled phase adjustments and baseline corrections within Chenomx. Spectra were transferred to the profiler within Chenomx and metabolites were assigned and quantified using the incorporated data bases for metabolite identification. Spectra were normalized on tissue weight for further statistical and functional analysis.

### Univariate statistical analysis

Univariate statistics were performed with SigmaPlot (v. 12.50, Systat Software, Inc, USA) and the results are presented as the mean ± standard error of the mean (SEM). Differences in plasma parameters and in liver metabolites between treatments at each time point were analysed by one-way analysis of variance (one-way ANOVA) on log2-transformed data, followed by the Holm-Šidák test. When transformed data failed the Shapiro-Wilk normality test, the Kruskal-Wallis one-way ANOVA on ranks was used followed by Dunn’s test. Two outlier samples were removed from the metabolome analysis. Adenosine triphosphate (ATP) was not considered because it did not vary between samples. The level of statistical significance was 5%.

Spearman rank correlation analysis with Bonferroni correction (i.e. statistical significance at p < 0.002) was performed between the log2 metabolite concentrations at the two response times. Box-and-whisker plots and heatmaps of metabolite correlations were generated in MetaboAnalyst v 3.0^44^.

### Multivariate statistical analysis

Principal Components Analysis (PCA) and Partial Least Squares – Discriminant Analysis (PLS-DA) were performed with MetaboAnalyst (v 3.0) in order to determine the global sample distribution in the metabolomics space and find global metabolic differences between the control and the hormone treatment groups. The metabolite concentrations were log2 transformed and mean-centered prior to PCA and PLS-DA. PLS-DA models were further validated by permutation testing using 2000 iterations to establish whether the observed discrimination between the groups was statistically significant (p < 0.05). Based on the PLS-DA model, variable importance in projection (VIP) scores were obtained, which were used to identify metabolites that had a large weight in the model and contributed significantly to the discrimination of samples from the different treatment groups.

## Results

### Blood plasma biochemistry

Hormonal treatments did not give a clear pattern of response in blood parameters (see Supplementary Fig. 1). At 6 h post injection, total calcium was significantly higher in the PTHrP(7–34) plus STC1A group compared to the PTHrP(7–34) and PTHrP(1–34) plus anti-STC1A groups. At 24 h, there were no statistically significant differences in total calcium between any of the groups. In contrast free calcium did not differ between groups at 6 h but at 24 h, levels were significantly lower in the PTHrP(7–34) group than in the control, PTHrP(1–34), and PTHrP(7–34) plus STC1 groups. At 6 h, total phosphorus was significantly lower in the PTHrP(7–34) group compared to the other groups. At 24 h, the levels of total phosphorus were significantly elevated in the PTHrP(1–34) plus anti-STC1 group compared to the control, PTHrP(1–34) and PTHrP(7–34) plus STC1A, while the PTHrP(7–34) group phosphorus was elevated only in relation to the latter two groups. At 6 h, plasma glucose in the PTHrP(1–34) group was significantly higher than the PTHrP(7–34) plus STC1A group and no differences were observed between the other groups. At 24 h, there were no differences in blood glucose between groups. Total protein, osmolality and lactate did not change in response to treatments (not shown).

### Metabolites modified by treatments

**Figure 1:**
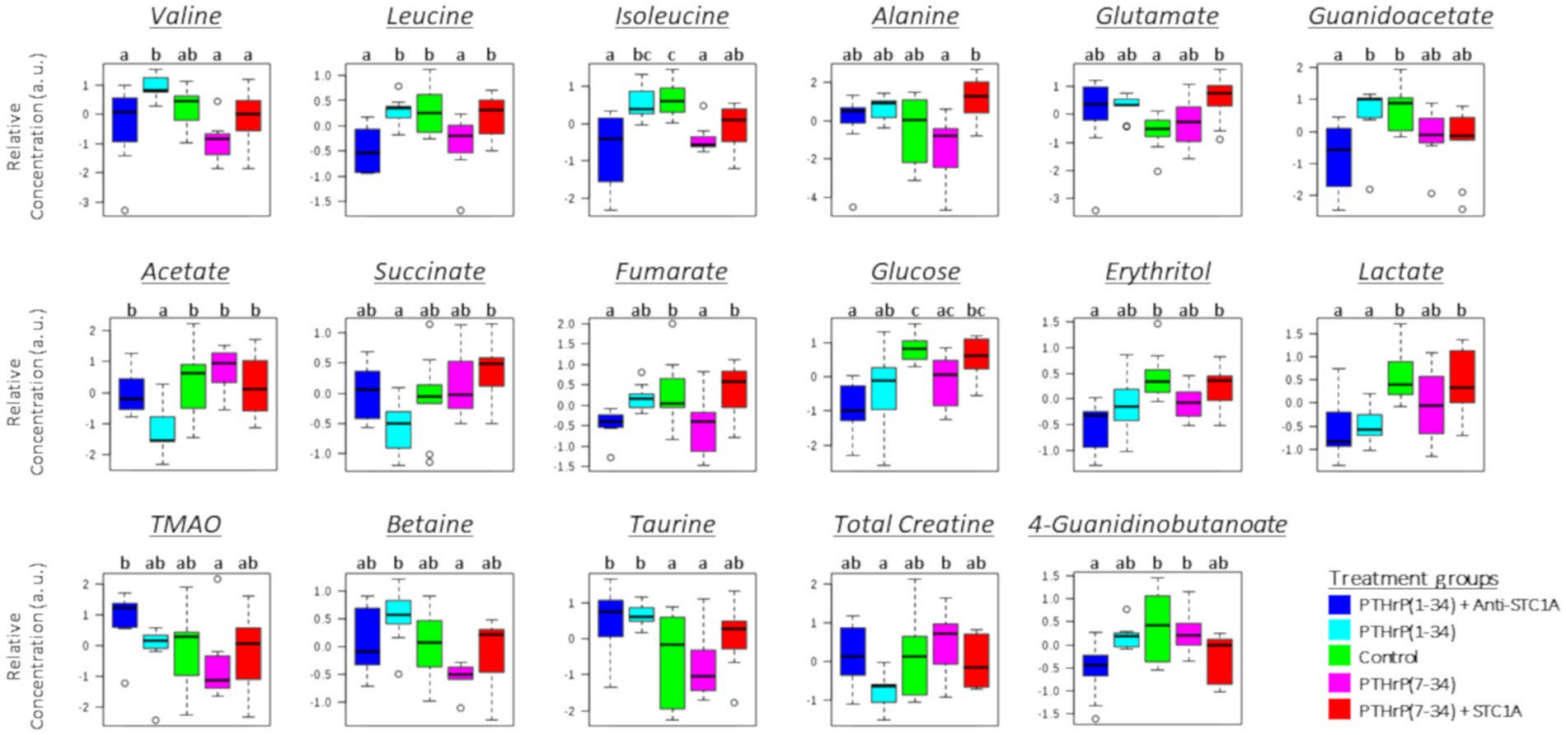
Box-and-whisker plots of relative concentrations for significantly changed metabolites in liver after 6 h of hormone treatment. Data were normalized and scaled and in the y axes the concentrations are represented as relative units. The control is green, PTHrP(1–34) is cyan, PTHrP(1–34) + Anti-STC1A is blue, PTHrP(7–34) is magenta and PTHrP(7–34) + STC1A is red. Different letters denote significant differences between groups (p < 0.05, one-way ANOVA).

**Figure 2:**
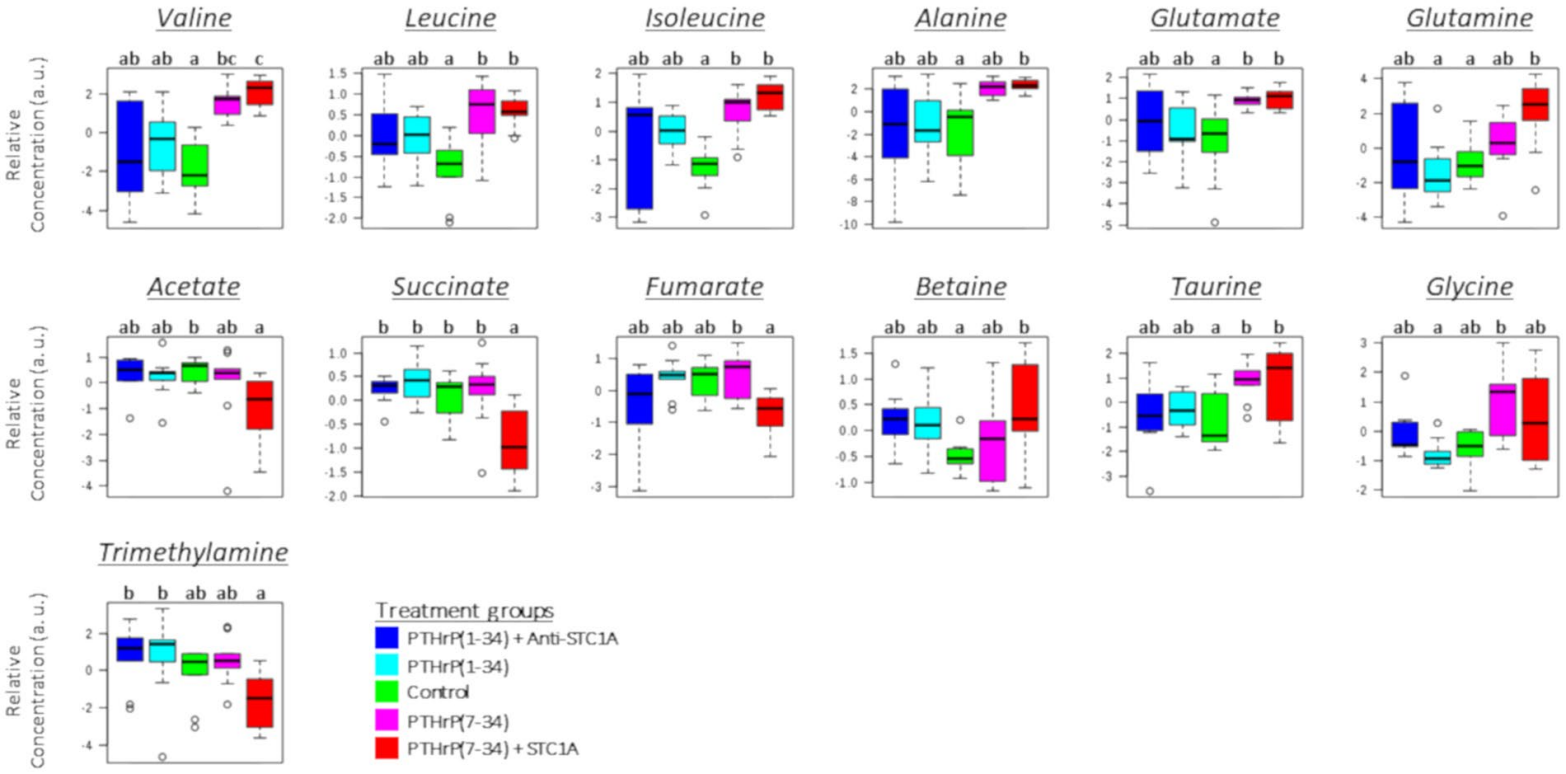
Box-and-whisker plots of relative concentrations for significantly changed metabolites in liver after 24 h of hormone treatment. Data were normalized and scaled as indicated in Fig. 1. The control is green, PTHrP(1–34) is cyan, PTHrP(1–34) + Anti-STC1A is blue, PTHrP(7–34) is magenta and PTHrP(7–34) + STC1A is red. Different letters denote significant differences between groups (p < 0.05, one-way ANOVA).

The ^1^H-NMR spectra of liver metabolites showed clear and reproducible profiles (Supplementary Fig. 2), with 26 metabolites identified and quantified, of which the concentrations of 17 and 13, respectively, at 6 h (Fig. 1) and 24 h post-injection (Fig. 2), differed significantly (p < 0.05) between treatment groups.

The metabolites displaying significant responses to treatments belonged to three main classes: amino acids, intermediates related to the citric acid cycle (CAC) and lipid metabolism. At 6 h, statistically significant responses compared to the control were found in the pro-PTHrP and in the PTHrP(7–34) groups (Fig. 1). However, at 24 h significant differences were found mainly in the pro-STC1 group treatments (Fig. 2). In addition, at 24 h the effect of hormone treatments seemed to be more evident than at 6 h.

At 6 h post-injection (Fig. 1), levels of BCAAs, namely valine, leucine and isoleucine were significantly elevated (p < 0.05) in the PTHrP(1–34) group compared to PTHrP(7–34). However, PTHrP(1–34) plus anti-STC1A had no effect or reduced the levels of BCAAs compared to the control, and PTHrP(7–34) plus STC1A had no clear effect. Similarly, PTHrP(1–34) and PTHrP(7–34) had significant (p < 0.05) but opposite effects on metabolites related to lipid metabolism (taurine and betaine), and on acetate, an intermediate related to the CAC. At 24 h post-injection (Fig. 2), the pro-STC1 treatments caused an increase in amino acids and metabolites related to lipid metabolism and a reduction in those related to the CAC. In addition, only the STC1 containing treatment caused a significant decrease in the levels of intermediates related to the CAC (acetate, succinate and fumarate) and trimethylamine (TMA).

### Correlation analysis of metabolites

**Figure 3:**
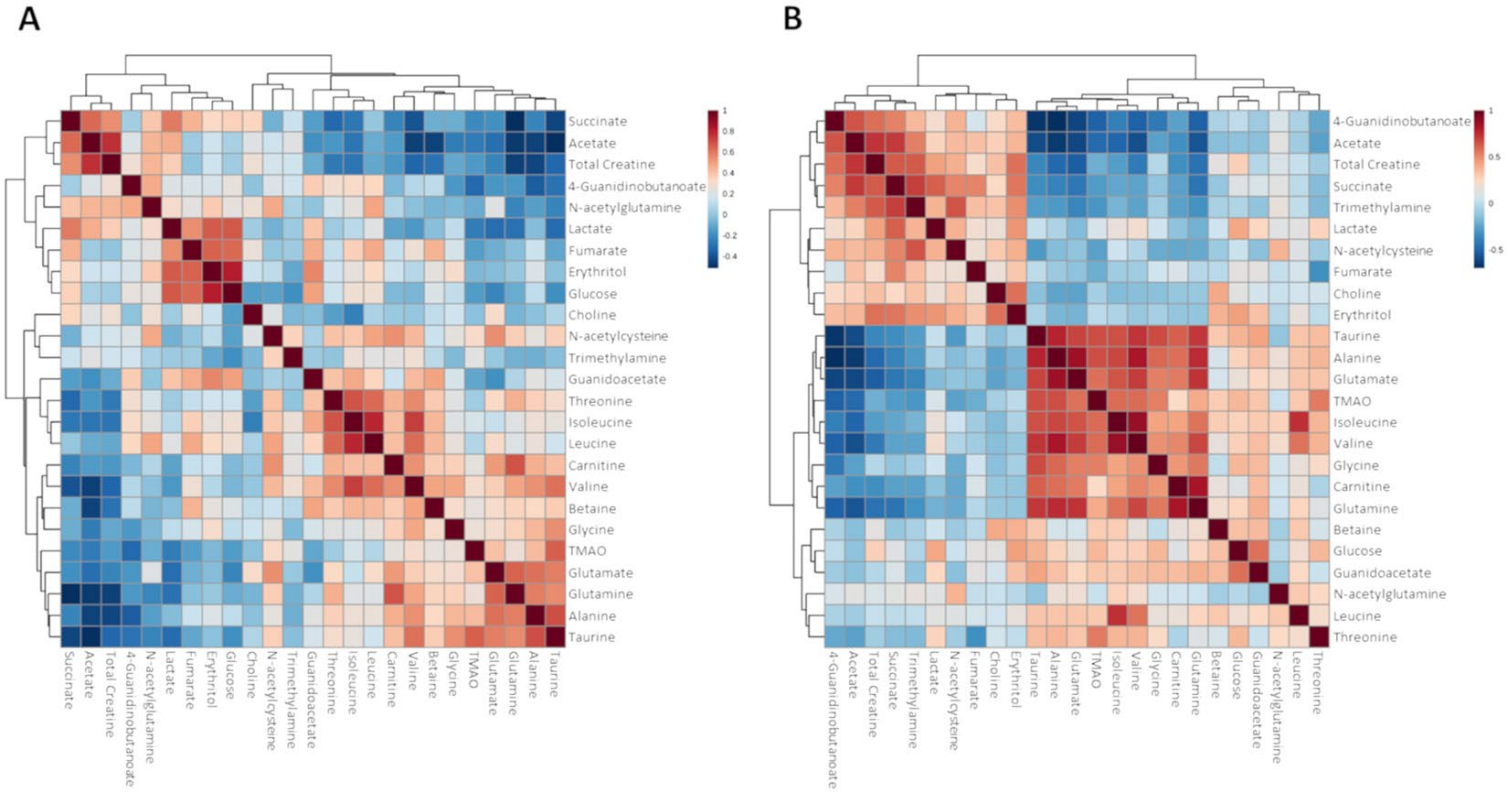
Correlogram of metabolites after 6 h (**A**) and 24 h (**B**) of treatment. Data of the relative concentration of metabolites were normalised and analysed using Spearman rank correlation analysis within MetaboAnalyst (v 3.0). Each coloured cell on the map indicates the correlation coefficient, with the scale code shown on the top right corner (red and blue colours mean positive and negative correlations, respectively).

A Spearman rank correlation analysis revealed two clusters, which displayed strong intra-cluster (positive) and inter-cluster (negative) correlations at 24 h post-injection relative to 6 h post-injection (Fig. 3A,B and Supplementary Tables 1A,B). Interestingly, one of the clusters contained mostly amino acids, while the other cluster contained mainly metabolites related to the CAC.

In the amino acid cluster at 6 h, alanine had a moderate to high positive correlation with valine, glutamine, glutamate and taurine. At 24 h, alanine had similar positive correlations with the same amino acids, as well as with isoleucine, glycine, trimethylamine N-oxide (TMAO) and carnitine, and was negatively correlated with acetate and 4-guanidinobutanoate. The BCAAs were positively correlated with each other at 6 h and 24 h. At 6 h, leucine and isoleucine were also positively correlated with threonine, while valine was positively correlated with threonine, as well as alanine, taurine and carnitine. At 24 h, isoleucine and valine were both positively correlated with alanine, taurine, glutamine, glutamate and TMAO. Additionally, valine was also positively correlated with carnitine and negatively correlated with acetate and 4-guanidinbutanoate.

In the CAC metabolites cluster, acetate was positively correlated with succinate and total creatine and negatively correlated with taurine at 6 h and 24 h. In addition, at 24 h acetate was positively correlated with TMA and 4-guanidinobutanoate, and negatively correlated with several amino acids including alanine, glutamate, glutamine and valine. Additionally, succinate was correlated positively with acetate, lactate and total creatine at both 6 h and 24 h post injection and negatively correlated with glutamine. At 24 h post injection, succinate was positively correlated with fumarate, 4-guanidinobutanoate, TMA, erythritol and N-acetylcysteine.

### Multivariate analysis and association of metabolite profiles with treatments

**Figure 4:**
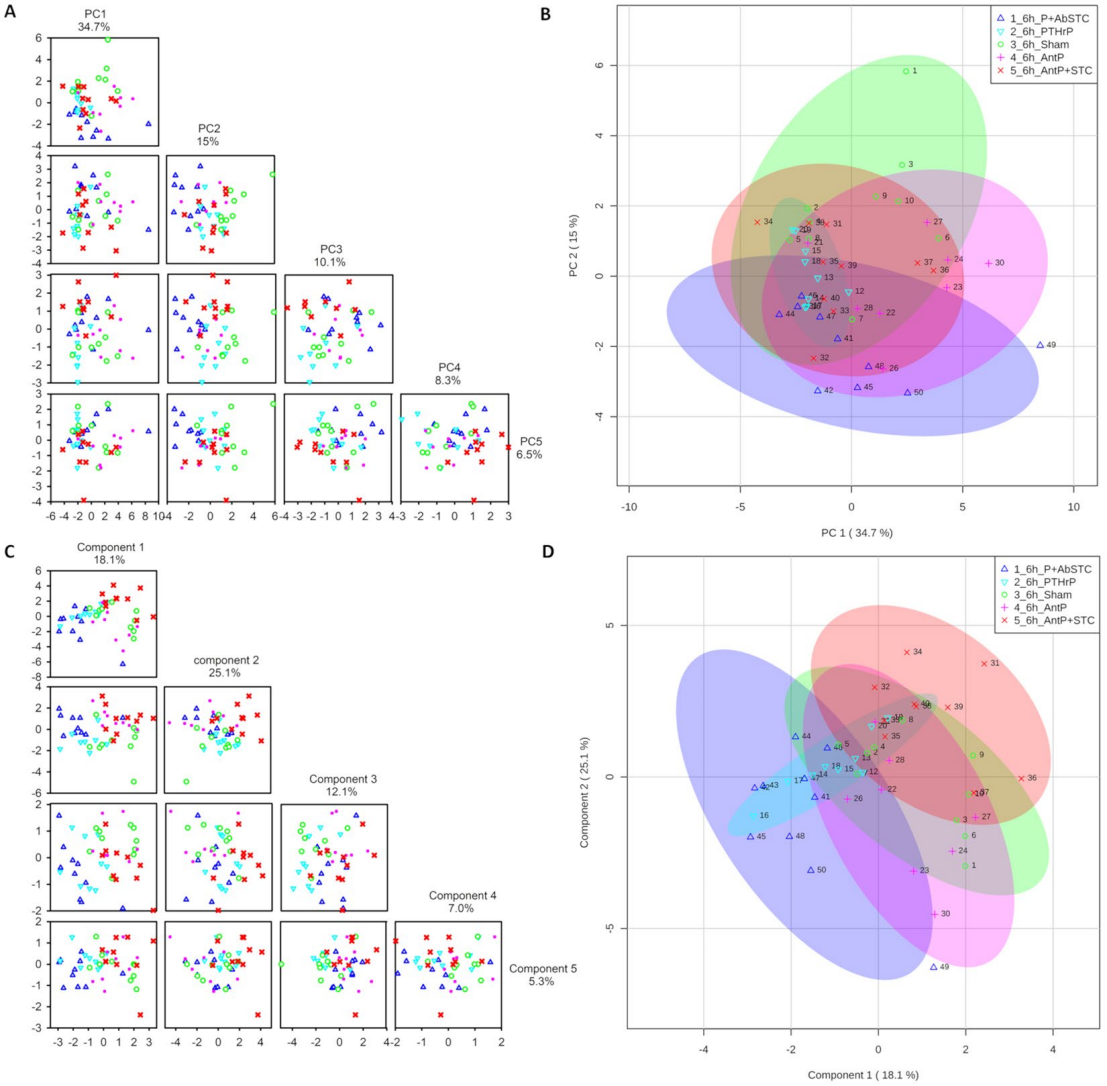
Multivariate analysis of metabolomics data 6 h post injection. (**A**) Principal component analysis (PCA). Pairwise score plots between the first five principal components of the PCA. The explained variance of each PC is shown in the corresponding diagonal cell. (**B**) PCA Score plot between the two first principal components. The explained variances are shown in the corresponding axes and the shaded areas indicate the 95% confidence regions. (**C**) Partial Least Squares – Discriminant analysis (PLS-DA). Pairwise score plots between the first five components of PLS-DA. The explained variance of each component is shown in the corresponding diagonal cell. (**D**) PLS-DA Score plot between the two first components. The explained variances are shown in the corresponding axes and the shaded areas indicate the 95% confidence regions. The experimental groups are the same and have the same colours as in Fig. 1.

**Figure 5:**
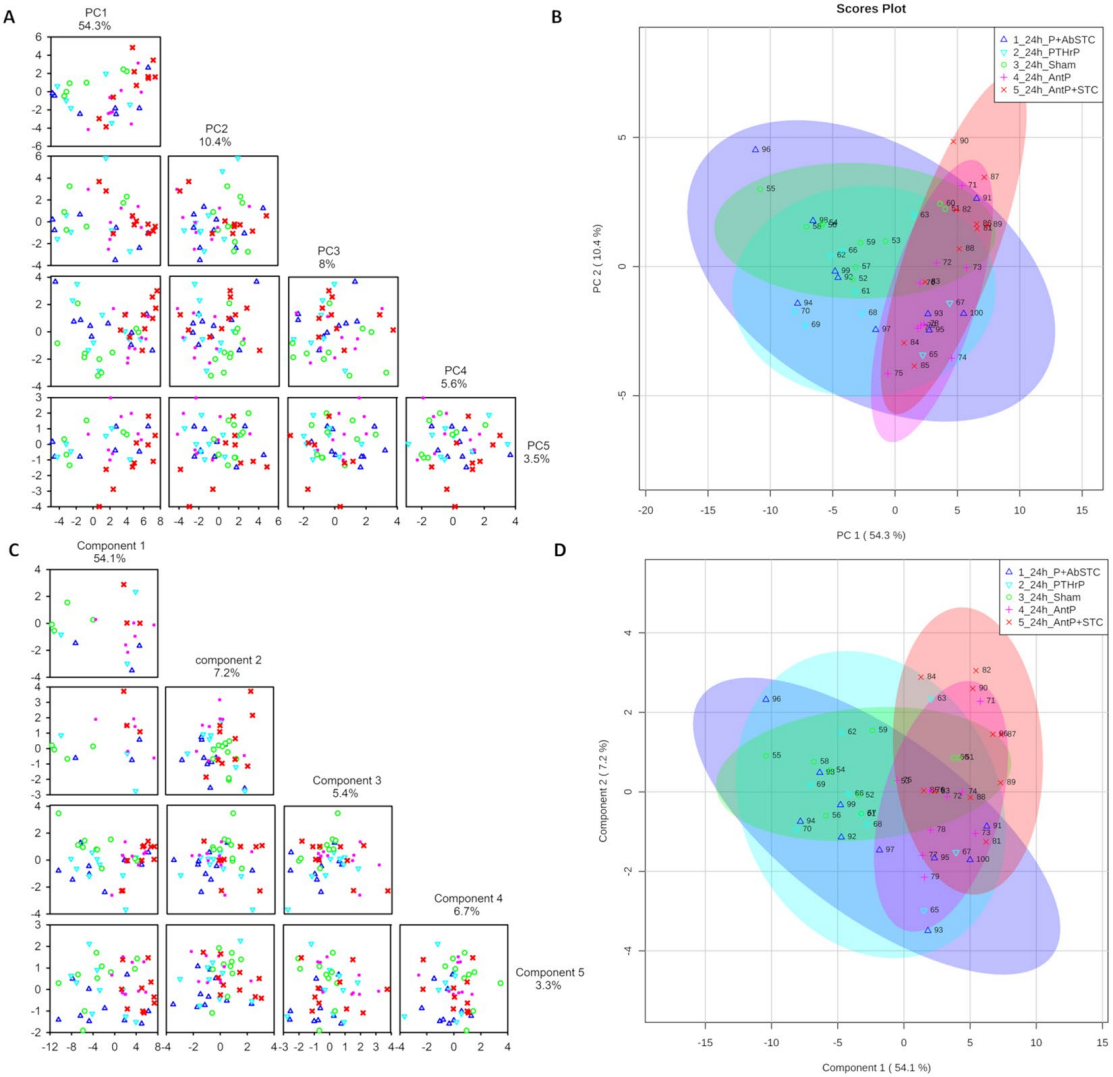
Multivariate analysis of metabolomics data 24 h post injection. (**A**) Principal component analysis (PCA). Pairwise score plots between the first five principal components of PCA. The explained variance of each PC is shown in the corresponding diagonal cell. (**B**) PCA Score plot between the two first principal components. The explained variances are shown in the corresponding axes and the shaded areas indicates the 95% confidence limits. (**C**) Partial Least Squares – Discriminant analysis (PLS-DA). Pairwise score plots between the first five components of PLS-DA. The explained variance of each component is shown in the corresponding diagonal cell. (**D**) PLS-DA Score plot between the two first components. The explained variances are shown in the corresponding axes and the shaded areas indicate the 95% confidence limits. The experimental groups are the same and have the same colours as in Fig. 1.

A PCA was performed on 25 metabolites separately at 6 h and 24 h. At 6 h, the two first principal components (PC) explained almost 50% of the total variation; 34.7% for PC1 and 15% for PC2 (Fig. 4A). At 24 h, the two first PC explained 64.7% of total variation; 54.3% for PC1 and 10.4% for PC2 (Fig. 5A). From the PCA score plots at 6 h (Fig. 4B) and 24 h (Fig. 5B), the differentiation between groups occurred mainly along PC1, and the largest source of variation were the experimental treatments. And, although the 95% confidence intervals generated for each treatment group overlapped in the PCA score plots, those with the same type of hormone treatment, i.e. the pro-PTHrP groups *vis a vis* the pro-STC1 groups, were closer together and aligned along the same axis, with a more pronounced separation at 24 h.

At 6 h, the PCA loadings with larger absolute weights on PC1 were acetate, total creatine, succinate, lactate, 4-guanidobutanoate, alanine, valine, taurine, glutamine and carnitine, while on PC2 they were guanidoacetate, glucose, threonine, TMAO, taurine and alanine (Supplementary Fig. 3A). At 24 h, the PCA loadings with larger absolute weights on PC1 were acetate, total creatine, succinate, 4-guanidobutanoate, alanine, valine, taurine, glutamine, glutamate and TMA, while on PC2 they were TMA, alanine, glutamine, carnitine, taurine and N-acetylcysteine (Supplementary Fig. 3B). Interestingly, there was a high overlap of the metabolites with higher absolute weights along PC1 (i.e. along the axis that better separates treatments) at 6 h and 24 h.

From the PLS-DA at 6 h and at 24 h (Figs 4C and 5C), the differentiation between groups occurred mainly along the first two components representing, respectively, 18.1% and 54.1% of the variance in component 1, and 25.1% and 7.2% in component 2 (Figs 4D and 5D). In the PLS-DA loading plot at 6 h, glucose, acetate, lactate, succinate, total creatine, TMAO, taurine, alanine, valine and glutamine were the most discriminant variables along component 1, while acetate, succinate, total creatine, alanine, glutamine and glutamate were the most discriminant variables along component 2 (Supplementary Fig. 4A). Thus, more than 50% of the variables with greater weight in component 1 of the PLS-DA were also selected by the PCA. Moreover, in the PLS-DA 24 h loading plot (Supplementary Fig. 4B), the metabolites displaying more weight in component 1 were exactly the same that contributed more to PC1 in the PCA, while of the metabolites displaying more weight in PLS-DA component 2 (4-guanidinobutanoate, TMAO, glycine, TMA, alanine and N-acetylcysteine), half coincided with those contributing more to the PC2. Thus, supervised and unsupervised multivariate analysis mainly gave higher weights to the same variables, supporting the notion that the hormone treatments had significant metabolic effects. In addition, as time elapsed the effects of hormones on metabolism became increasingly clear, since at 24 h the variables that better explained the variation obtained by PCA and PLS-DA were exactly the same and in the same order of absolute weight. Additionally, the separation achieved by PLS-DA was validated by permutation testing (2000 iterations; p < 0.0005 at 6 h or 24 h) indicating that the separation achieved was unlikely to have occurred by chance.

**Figure 6:**
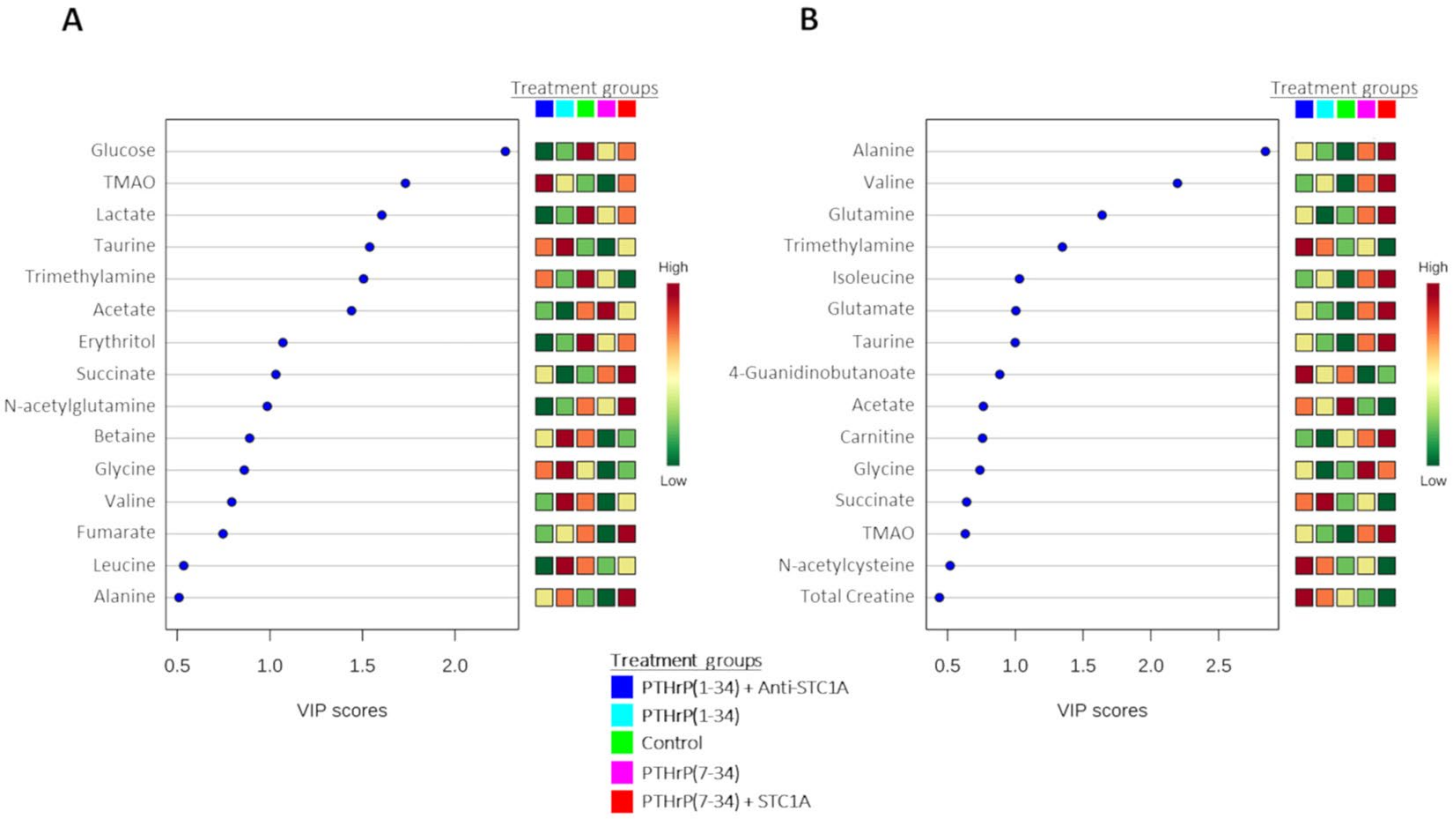
Important features identified by PLS-DA and VIP scores at 6 h (**A**) and at 24 h (**B**) of hormone treatments. The TOP 15 important features which correspond to the metabolites are indicated on the left and are organized by descending order of value of variable importance in projection (VIP) score in component 1. The coloured boxes on the right indicate the relative concentrations of the corresponding metabolite in each group under study. The relative concentration of metabolites is represented by a coloured scale from green to red indicating the low and high, respectively. The experimental treatment groups are the same and have the same colours as in Fig. 1.

The important features identified by the PLS-DA and the VIP score plot for component 1 at 6 h (18.1% discriminant ability; Fig. 6A) and at 24 h (54.1% discriminant ability; Fig. 6B) include the 15 key metabolites that better explained the model variation and were in accordance with the univariate and multivariate PCA analyses - at 24 h the metabolites with the highest VIP scores coincided with those found in the univariate analysis and the PCA. At 6 h and 24 h the prominent metabolites identified were intermediates of the CAC and lipid metabolism, while at 24 h they also include the amino acids alanine, glutamine, glutamate and the BCAAs. At 24 h, in the PTHrP(7–34) group amino acids were elevated and in the PTHrP(7–34) plus STC1 they were higher still.

## Discussion

The present study demonstrates that STC1 and PTHrP, the two major calciotropic hormones in fish, strongly modulate the liver concentration of metabolites related to gluconeogenesis and lipid metabolism.

### Pro-STC1 treatments increased the levels of amino acids

At 24 h post-injection, pro-STC1 treatments caused a strong and significant increase in the concentration of amino acids in liver, namely alanine, valine, leucine, isoleucine, glutamine and glutamate, which are strongly associated with energy metabolism. Alanine is a major glucogenic precursor and an important energy substrate in fish, while glutamine, glutamate and the BCAA are also involved in glucose regeneration and feed the CAC^45–48^. Interestingly, these amino acids are also involved in muscle and liver energetics^47–49^, and this is consistent with the suggestion that STC1 causes mobilization of amino acids from muscle protein to the liver for energy production. Previous studies have shown that fishes in food deprivation conditions have different metabolic responses in the usage/prioritize energy resources, some use in first place the fat and glycogen reserves with conservation of the muscle proteins and others use primarily the muscle amino acids as fuel for gluconeogenesis with the conservation of liver glycogen content^50–54^. Additionally, it was suggested that the selection of the metabolic fuel could be dependent of exogenous and endogenous factors such as hormones^52^. In carnivorous fishes, like European sea bass, the muscle is the main storage tissue for utilizable proteins and through proteolysis the released amino acids can either be oxidized for energy production or converted to other utilizable forms by anabolic processes, such gluconeogenesis^55,56^. Since our fish were up to 48 h without feeding (24 h prior and 24 h during the experiment), the increase in some essential amino acids in the pro-STC1 treatment groups, indicates their mobilization from muscle proteolysis. Additionally, the mobilization of alanine from muscle to liver, originated through the transamination of glutamate and pyruvate to form alanine, is a source of energy and avoids ammonium release, which is toxic^45,55^.

Thus, it is hypothesized that under STC1 stimulation, amino acids are prioritized as energy resources, and consequently, alanine from muscle protein breakdown is blood transported to the liver, where it is converted to pyruvate by alanine aminotransferase, which in turn can be used for gluconeogenesis^46,48,50,57^, a process with similarities to the glucose-alanine cycle in mammals^48,58^. In addition, pro-STC1 treatments increased significantly the concentration of glutamine and glutamate which can enter the CAC or can be used for gluconeogenesis. This is in line with a recent report showing increased STC1-stimulated gluconeogenesis from ^14^C-glutamine in sea bass kidney slices^22^. The significant increase in BCAAs in sea bass liver 24 h after the pro-STC1 treatment, possibly also originated from muscle. In contrast to mammals, fishes have little white muscle BCAA aminotransferase activity^49,59^ and it has been proposed that the fish liver has a greater role in BCAA metabolism for energy production^46^. Our hypothesis is further supported by observations that (1) STC1 transgenic mice had higher glucose clearance and increased myocytes mitochondria size than their wild type litter mates^13^; (2) the liver is the main organ for amino acid catabolism^60^; and (3) amino acid metabolism in skeletal muscle is primarily for generation of ATP as an immediate source of energy^45^.

Glutamate and glutamine, besides being gluconeogenic substrates, also participate in the ammonia detoxification system, and glutamine synthesis has been reported as a strategy for ammonia detoxification^61^. Indeed, glutamine synthesis is abundant in several tissues, including liver, and the corresponding enzyme is up-regulated by high environmental ammonia^62^, which in turn is also related to an increase of protein catabolism^55^. Taken together, these results lend further support our hypothesis, that under STC1 stimulation amino acids derived from muscle protein breakdown are strategically prioritized as metabolic fuels and are mobilized to the liver for energy production. This strategy minimizes ammonia production and accumulation, while safeguarding glycogen stores that can be used in stress situations.

### Pro-STC1 treatments decreased the levels of CAC intermediates

PTHrP(7–34) plus STC1 treatment caused a significant decrease in the CAC intermediates, succinate, fumarate and acetate, in the liver at 24 h post-injection. A likely explanation for this is that the process of conversion of alanine to pyruvate (to allow gluconeogenesis) is dependent of oxaloacetate usage^45,46^, which consequently decreases the availability of intermediates of CAC. In addition, the CAC was possibly modified due to low concentrations of intermediates. Alternatively, if the amino acids were being converted into glucose (gluconeogenesis) to be exported to peripheral tissues, the resulting liver glucose scarcity could inhibit the CAC^63^. However, in the present study liver glucose levels did not change significantly with pro-STC1 treatments. Additionally, alanine is known to be a regulator of glycolysis through modulation of pyruvate kinase, the last enzyme of the pathway leading to pyruvate^63^. Thus, in a situation where alanine levels are elevated (as with the pro-STC1 treatments), this could result in the stimulation of gluconeogenesis and the inhibition of glycolysis, which in turn could inhibit the CAC.

Fish, unlike mammals, have a limited ability to metabolize glucose, even though they have all the enzymatic machinery necessary for carbohydrate utilization^64^. It is therefore possible that STC1 has a regulatory effect on glycolysis, gluconeogenesis and even on the CAC by increasing the availability of amino acids in the liver. However, more studies focusing on the intermediate metabolism are needed to confirm this aspect.

### Pro-STC1 and Pro-PTHrP treatments modify lipid metabolism in opposing directions

Pro-STC1 treatments at 24 h significantly increased the levels of metabolites related to lipid metabolism, namely taurine and betaine. Taurine is an aminosulfonic acid that acts as an osmolyte to maintain cellular homeostasis and in bile acid conjugation^65,66^. Betaine is a methyl donor and an osmolyte that results from the degradation of choline^66^. Furthermore, multivariate discriminant analysis revealed that Pro-STC1 treatments also increased carnitine and TMAO, while both univariate and multivariate analysis showed that TMA levels decreased, all of which metabolites are related to lipid metabolism^66,67^. Thus, STC1 seems to promote lipogenesis and lipid accumulation. This hypothesis is supported by the fact that TMA can be converted to TMAO, which in turn inhibits the β-oxidation of lipids and promotes lipid storage^68,69^. Consistent with our results, it has been recently shown *in vitro* studies with fed rats that STC1 increases lipogenesis^23^. This will be an important area to explore in future studies.

Pro-PTHrP treatments increased significantly the levels of taurine at 6 h but by 24 h they were significantly reduced. In addition, multivariate discriminant analysis revealed that the concentrations of TMAO and carnitine decreased and TMA increased in the pro-PTHrP groups. This indicates that PTHrP affects lipid metabolism in the opposite direction to STC1 and stimulates lipolysis and β-oxidation of lipids. PTHrP has been shown to modify the composition and turnover of phospholipids in membranes^35^, while PTH(1–34) activates lipolysis in human adipose tissue *in vitro* and its antagonist, PTH(3–34) counteracts this effect by competing at the level of the PTH receptor^38^. A recent study with mouse adipocytes revealed that PTH(1–84), as well as PTH(1–34), induced lipolysis via protein kinase A mediated phosphorylation of hormone-sensitive lipase^39^. Based on these and our own results it appears that the effect of PTH/PTHrP on lipid metabolism may have been conserved in vertebrates.

### Pro-PTHrP treatments decreased the levels of sugars and lactate

Pro-PTHrP treatments significantly decreased the liver levels of glucose and erythritol as well as lactate within 6 h. Since lactate is a dead-end metabolite produced in muscle^70^ this may indicate that PTHrP promotes their utilization in gluconeogenesis. Interestingly, studies in tetrapods reported that PTH, which shares a common receptor with PTHrP, promotes renal gluconeogenesis from lactate^36^ and liver gluconeogenesis from alanine^37^. Overall, it appears that PTHrP has a role in the regulation of gluconeogenesis in vertebrates including the European sea bass and presumably also promotes hepatic glucose export to peripheral tissues.

### Time-dependent responses of PTHrP and STC

It appears that pro-PTHrP and pro-STC1 treatments act over different time scales on metabolism and the former seemed to have more pronounced effects at 6 h, while the latter had larger effects 24 h after treatment. This may be a consequence of differences in absorption, circulation in the blood and turnover time due to their differing molecular weights and chemical properties, which may affect their *in vivo* pharmacokinetics. Interestingly, blocking endogenous hormones with anti-STC1A serum showed effects mainly at 6 h after treatment while for the PTHrP(7–34) antagonist effects were seen at 24 h. This could also suggest different mechanisms of action and/or target tissues. Support for this hypothesis comes from studies that related the action of this hormone mediated by its effect on cyclic adenosine monophosphate (cAMP), on amino acid metabolism and gluconeogenesis^71^ and on lipid metabolism^72^. Moreover, it was also documented that PTHrP activates cAMP production and accumulation^73^, while STC1 has an inhibitory effect on cAMP accumulation^74^.

### Lack of a pattern of calcium and phosphate responses

The effects of the treatments on metabolism were apparent even though no clear responses in plasma calcium or phosphate were observed. This may suggest that the effect on metabolism is independent from the ionic responses, but specific studies are required to clarify this issue. We do not have a good explanation for the lack of effects on plasma calcium and phosphate, but this may be linked to the fact that these ions are tightly regulated. For example, it is possible that the direct action of the treatments on calcium may have manifested sooner, and the results obtained may reflect a suite of compensatory processes – while at 6 h most small changes occur on protein bound calcium, keeping free levels stable, at 24 h smaller variations occur only in free calcium. The compensatory responses of calcium-regulatory mechanism are elusive, and often the calciotropic actions can only be perceived by determining calcium entry (influx) or excretion (efflux) using radiotracers, and not by actual changes in circulating level^75^. Thus, the reasons for the calcium results could be several: timing of response, temperature previous history, species specificity vs dosage, endogenous levels, including compensatory responses^76^.

### Concluding remarks

**Figure 7:**
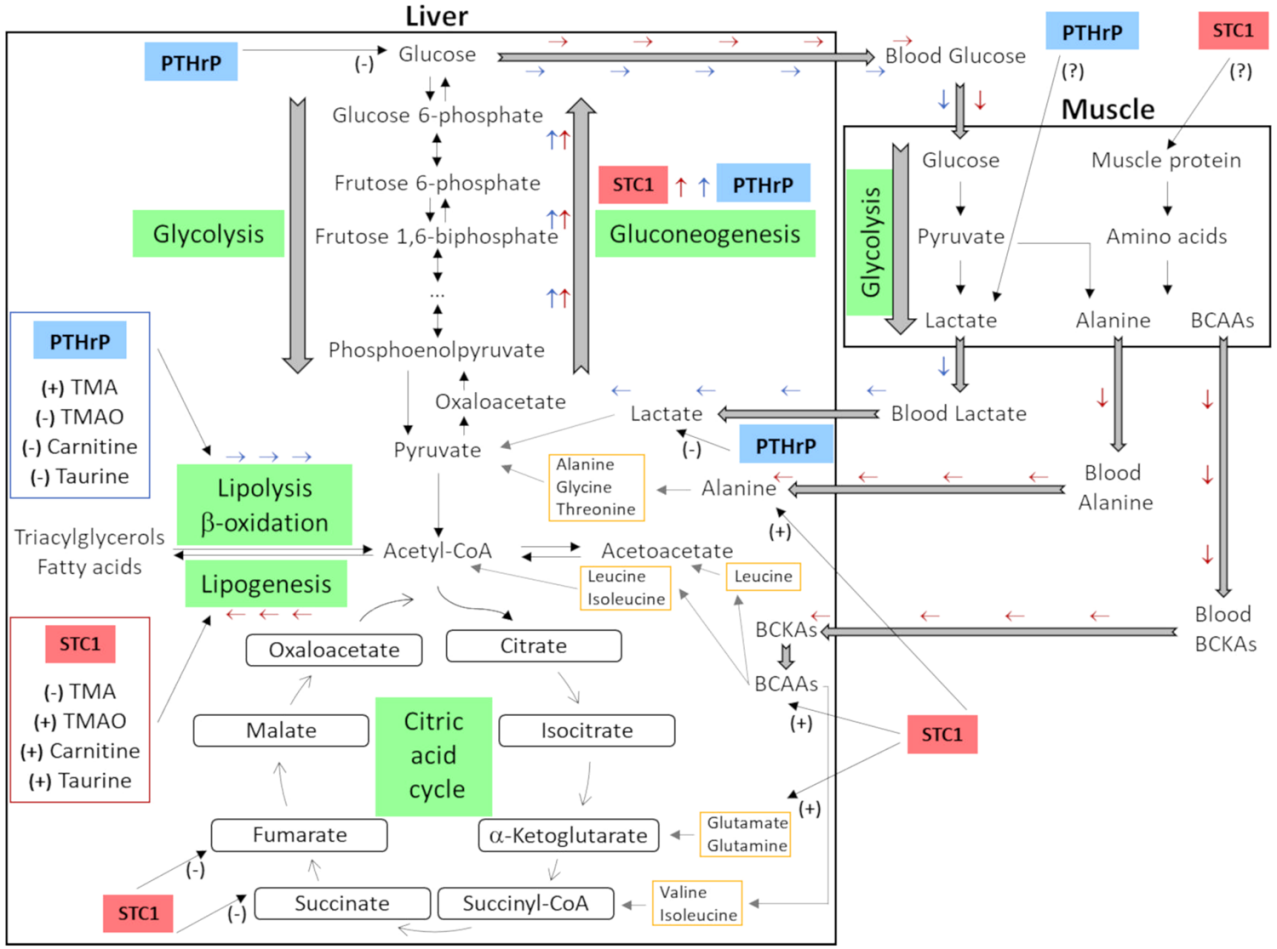
Schematic representation of the metabolic pathways modulated by STC1 and PTHrP. STC1 increased the liver concentration of branched-chain amino acids (valine, leucine and isoleucine), alanine, glutamine and glutamate, suggesting mobilization of amino acids from muscle to liver to produce glucose through gluconeogenesis. PTHrP decreased the liver concentrations of glucose and lactate suggesting a stimulatory effect on gluconeogenesis. STC1 and PTHrP had an opposing effect on the liver concentration of TMA, TMAO, carnitine and taurine suggesting that STC1 stimulates lipogenesis and PTHrP activates lipolysis/fatty acids β-oxidation. The names of the metabolic pathways are highlighted in green and the effects of the hormones STC1 and PTHrP are highlighted in red and blue, respectively. The amino acids listed in the yellow boxes correspond to the amino acids identified in this study, which are glucogenic and ketogenic and can be converted into intermediates of the CAC. Red arrows and blue arrows indicate, respectively, the metabolic pathways modulated by STC1 and PTHrP. (+) indicates an increase while (−) indicates a decrease in the concentration of metabolites in the liver caused by the hormone treatments. BCKAs: branched-chain alpha-keto acids.

STC1 and PTHrP promoted gluconeogenesis from different substrates and had antagonistic effects on the regulation of lipid metabolism in the European sea bass liver. We propose that the findings of the present study reveal a steady-state balance between the functions of the two hormones, which are related to strategic energy mechanisms that involve the production of glucose and consequently safeguard the liver glycogen reserves for stressful situations as depicted in the model outlined in Fig. 7. PTHrP acts faster than STC1 and decreased the concentration of sugars and lactate, suggesting it stimulates gluconeogenesis from lactate. STC1 then elevated amino acid concentrations, particularly the BCAAs, alanine, glutamine and glutamate and this suggests it mobilizes amino acids from muscle to liver to produce glucose through gluconeogenesis or feeds the CAC by degradation of the BCAAs. Both STC1 and PTHrP affected compounds related to lipid metabolism and the opposing effect of the two hormones on TMA, TMAO, taurine and carnitine concentrations indicate antagonistic effects on lipid metabolism. Thus, while STC1 stimulates lipogenesis, PTHrP activates lipolysis (Fig. 7), possibly via different G-proteins (e.g., Gα or Gαi) and activation/inhibition of cAMP.

Future studies will target the transcriptomic changes associated with the actions of these hormones in liver metabolism.

## Electronic supplementary material

Supplementary Information

## Data Availability

The raw data of the results presented in the paper are available upon request to the corresponding author.
