# Supplementary material for: STC1 and PTHrP Modify Carbohydrate and Lipid Metabolism in Liver of a Teleost Fish
Source: Sci Rep. 2019 Jan 24;9:723. doi: 10.1038/s41598-018-36821-2 (PMC6346029; doi:10.1038/s41598-018-36821-2)
Supplement: Supplementary file 1 — Supplementary Information [file 41598_2018_36821_MOESM1_ESM.pdf]

Supplementary Information

STC1 and PTHrP Modify Carbohydrate and Lipid Metabolism in Liver of a Teleost Fish

Short title: Metabolic effects of STC1 and PTHrP

Pedro F. S. Palma[1], Christian Bock[2], Tomé S. Silva[3], Pedro M. Guerreiro[1], Deborah M. Power[1], Hans-Otto Pörtner[2] and Adelino V. M. Canário[1$]

[1] CCMAR/CIMAR – Centre of Marine Sciences, University of Algarve, Campus de Gambelas, 8005-139 Faro, Portugal

[2] AWI – Integrative Ecophysiology, Alfred-Wegener-Institute Helmholtz Centre for Polar and Marine Research, Am Handelshafen 12, 27570 Bremerhaven, Germany

[3] SPAROS, Lda., Área Empresarial de Marim, Lote C, 8700-221, Olhão, Portugal

[$] **Corresponding author:** Adelino V. M. Canário, E-mail: acanario@ualg.pt

To whom reprints should be addressed: Adelino V. M. Canário, CCMAR/CIMAR Centre of Marine Sciences, University of Algarve, Campus de Gambelas, 8005-139 Faro, Portugal, E-mail: acanario@ualg.pt

Supplementary Table 1A: Spearman rank correlation analysis data of metabolites in the liver of sea bass treated with hormones at 6 h post-injection. For each correlation the first cell has the correlation coefficient and the cell below the p-value.

	Acetate	Total Creatine	4-Guanidinobutanoate	N-acetylglutamine	Lactate	Fumarate	Erythritol	Glucose	Choline	N-acetylcysteine	Trimethylamine	Guanidoacetate	Threonine	Isoleucine	Leucine	Carnitine	Valine	Betaine	Glycine	TMAO	Glutamate	Glutamine	Alanine	Taurine
**Succinate**	0.639	0.55	0.0385	0.385	0.593	0.446	0.326	0.354	0.342	-0.0715	0.148	-0.136	-0.327	-0.277	0.00463	-0.23	-0.397	-0.0826	-0.0834	-0.206	-0.179	-0.512	-0.226	-0.443
	0.00000113	0.0000717	0.796	0.00779	0.0000128	0.00177	0.0256	0.0148	0.0189	0.631	0.32	0.359	0.0251	0.059	0.975	0.119	0.00594	0.579	0.575	0.165	0.228	0.000263	0.126	0.00196
**Acetate**		0.775	0.201	0.441	0.464	0.0241	0.152	0.0494	0.21	0.15	0.218	-0.178	-0.161	-0.282	-0.0905	-0.143	-0.461	-0.451	-0.255	-0.263	-0.305	-0.476	-0.451	-0.514
		0.0000002	0.175	0.00206	0.0011	0.872	0.306	0.74	0.155	0.313	0.14	0.231	0.277	0.0552	0.544	0.335	0.0012	0.00159	0.0832	0.0744	0.0375	0.000795	0.00157	0.000243
**TotalCreatine**			0.277	0.439	0.365	-0.00931	0.152	0.0302	0.272	0.175	0.155	-0.093	-0.272	-0.278	-0.102	-0.158	-0.338	-0.295	-0.104	-0.148	-0.226	-0.46	-0.44	-0.332
			0.0599	0.00214	0.012	0.95	0.306	0.839	0.0642	0.238	0.297	0.532	0.0644	0.0582	0.493	0.288	0.0205	0.044	0.485	0.319	0.127	0.00124	0.00208	0.0229
**4-Guanidinobutanoate**				0.472	0.203	0.194	0.187	0.238	-0.012	0.198	0.165	0.368	0.288	0.313	0.337	-0.0477	0.0864	0.11	-0.156	-0.331	-0.164	-0.155	-0.367	-0.294
				0.000889	0.171	0.19	0.207	0.106	0.935	0.181	0.266	0.0111	0.0496	0.0325	0.0207	0.749	0.562	0.461	0.293	0.0235	0.27	0.298	0.0114	0.0451
**N-acetylglutamine**					0.298	0.226	0.374	0.241	0.267	0.475	0.0592	-0.00792	0.183	0.235	0.487	0.0775	-0.0191	-0.063	-0.0473	-0.0914	0.208	-0.219	-0.133	-0.229
					0.0419	0.126	0.00993	0.103	0.07	0.0008	0.691	0.957	0.217	0.111	0.000567	0.603	0.898	0.672	0.751	0.539	0.16	0.138	0.37	0.121
**Lactate**						0.545	0.67	0.679	0.0306	-0.0752	0.0197	0.392	0.0292	0.0997	0.213	-0.124	-0.157	-0.0287	0.116	-0.254	-0.312	-0.3	-0.0295	-0.317
						0.000086	7.68E-09	0.0000002	0.837	0.614	0.894	0.00661	0.845	0.503	0.149	0.406	0.289	0.847	0.434	0.0842	0.0327	0.0407	0.843	0.0303
**Fumarate**							0.656	0.633	0.254	0.00602	0.0295	0.463	0.127	0.357	0.475	0.134	0.267	0.448	0.183	-0.137	-0.0807	-0.027	0.144	-0.112
							2.66E-07	1.56E-06	0.0842	0.968	0.843	0.00112	0.392	0.0139	0.000803	0.368	0.0695	0.0017	0.216	0.355	0.588	0.856	0.334	0.453
**Erythritol**								0.818	0.146	0.109	-0.106	0.565	0.164	0.239	0.323	0.152	0.0862	0.234	0.304	-0.0472	-0.0299	0.0105	0.201	-0.0204
								0.0000002	0.327	0.465	0.477	0.0000408	0.268	0.105	0.027	0.308	0.563	0.113	0.0378	0.752	0.841	0.944	0.175	0.891
**Glucose**									-0.139	-0.126	-0.185	0.5	0.151	0.276	0.273	-0.0433	-0.0533	0.199	0.122	-0.147	-0.223	-0.0896	0.114	-0.188
									0.351	0.396	0.212	0.00038	0.309	0.0602	0.0637	0.771	0.721	0.178	0.414	0.321	0.131	0.547	0.444	0.204
**Choline**										0.211	-0.0472	-0.0317	-0.13	-0.245	0.0611	0.0168	0.00613	0.117	0.16	-0.0191	0.305	-0.0612	-0.0186	0.0726
										0.154	0.752	0.831	0.382	0.0966	0.682	0.91	0.967	0.433	0.283	0.897	0.0374	0.682	0.9	0.626
**N-acetylcysteine**											0.348	0.0487	0.401	0.385	0.496	0.543	0.429	0.145	0.235	0.31	0.545	0.367	0.235	0.357
											0.0167	0.744	0.00544	0.00783	0.000437	0.0000923	0.00277	0.328	0.111	0.0344	0.0000867	0.0115	0.112	0.014
**Trimethylamine**												0.0213	-0.00727	0.265	0.222	0.226	0.276	0.0983	-0.0436	0.225	0.00862	-0.0719	-0.0789	-0.0276
												0.886	0.961	0.0711	0.132	0.126	0.0603	0.509	0.77	0.128	0.953	0.629	0.597	0.853
**Guanidoacetate**													0.4	0.475	0.339	0.154	0.422	0.496	0.18	-0.109	-0.18	0.0727	0.22	0.182
													0.00551	0.000803	0.0199	0.3	0.00332	0.000432	0.225	0.464	0.225	0.625	0.137	0.219
**Threonine**														0.69	0.635	0.447	0.562	0.377	0.218	0.0349	0.32	0.459	0.361	0.302
														0.0000002	1.37E-06	0.00175	0.0000456	0.00933	0.141	0.815	0.0286	0.00126	0.013	0.0394
**Isoleucine**															0.797	0.413	0.735	0.438	0.209	0.182	0.131	0.215	0.268	0.242
															0.0000002	0.00407	0.0000002	0.00222	0.157	0.22	0.378	0.146	0.0688	0.101
**Leucine**																0.411	0.632	0.427	0.245	0.0823	0.311	0.205	0.26	0.156
																0.00427	1.68E-06	0.00293	0.0962	0.581	0.0337	0.165	0.0769	0.293
**Carnitine**																	0.543	0.371	0.357	0.233	0.517	0.687	0.459	0.413
																	0.0000919	0.0104	0.014	0.115	0.000225	0.0000002	0.00125	0.00404
**Valine**																		0.492	0.482	0.3	0.362	0.486	0.544	0.621
																		0.000492	0.000661	0.0403	0.0126	0.000579	0.0000893	3.25E-06
**Betaine**																			0.306	0.239	0.305	0.347	0.327	0.373
																			0.0366	0.106	0.037	0.0171	0.0252	0.0101
**Glycine**																				0.265	0.293	0.351	0.433	0.55
																				0.0722	0.0458	0.0157	0.00253	0.000072
**TMAO**																					0.405	0.264	0.42	0.659
																					0.00494	0.0726	0.00342	1.89E-07
**Glutamate**																						0.655	0.618	0.59
																						3.18E-07	3.68E-06	0.000014
**Glutamine**																							0.591	0.551
																							0.000014	0.0000679
**Alanine**																								0.702
																								0.0000002
**Taurine**																								

Supplementary Table 1B: Spearman rank correlation analysis data of metabolites in the liver of sea bass treated with hormones at 24 h post-injection. For each correlation the first cell has the correlation coefficient and the cell below the p-value.

	Acetate	Total Creatine	Succinate	Trimethylamine	Lactate	N-acetylcysteine	Fumarate	Choline	Erythritol	Taurine	Alanine	Glutamate	TMAO	Isoleucine	Valine	Glycine	Carnitine	Glutamine	Betaine	Glucose	Guanidoacetate	N-acetylglutamine	Leucine	Threonine
**4-Guanidinobutanoate**	0.655	0.577	0.525	0.359	0.206	0.383	0.0745	0.236	0.344	-0.666	-0.696	-0.611	-0.516	-0.394	-0.515	-0.421	-0.265	-0.514	-0.0488	0.00825	-0.0886	0.0857	-0.0531	-0.236
	0.00000021	0.0000206	0.000143	0.0124	0.159	0.00736	0.613	0.106	0.0169	3.34E-08	0.0000002	0.00000417	0.000196	0.0058	0.000201	0.00302	0.0691	0.000209	0.74	0.955	0.547	0.561	0.719	0.106
**Acetate**		0.73	0.72	0.531	0.268	0.357	0.333	0.28	0.375	-0.591	-0.653	-0.604	-0.483	-0.498	-0.579	-0.262	-0.323	-0.558	-0.151	-0.135	-0.145	0.129	-0.0822	-0.262
		0.0000002	0.0000002	0.000119	0.0653	0.013	0.0209	0.0541	0.00879	0.0000111	0.00000026	0.00000594	0.000557	0.000357	0.0000184	0.0717	0.0256	0.0000444	0.306	0.357	0.323	0.38	0.577	0.0723
**TotalCreatine**			0.635	0.633	0.438	0.391	0.222	0.337	0.572	-0.363	-0.49	-0.531	-0.211	-0.274	-0.413	-0.0265	-0.33	-0.447	0.136	0.28	-0.022	0.0971	0.0322	-0.0851
			0.00000104	0.0000012	0.00196	0.00622	0.128	0.0193	0.0000253	0.0115	0.000456	0.00012	0.15	0.0594	0.00368	0.857	0.0224	0.00157	0.355	0.0541	0.881	0.51	0.827	0.564
**Succinate**				0.707	0.619	0.521	0.522	0.25	0.558	-0.334	-0.385	-0.43	-0.281	-0.243	-0.256	-0.127	-0.26	-0.441	-0.116	0.115	0.0961	0.129	-0.000109	-0.155
				0.0000002	0.00000271	0.000166	0.000163	0.0865	0.0000444	0.0207	0.00712	0.00243	0.0535	0.096	0.0785	0.388	0.074	0.00179	0.43	0.434	0.514	0.381	0.999	0.291
**Trimethylamine**					0.379	0.65	0.338	0.325	0.496	-0.275	-0.287	-0.368	-0.301	-0.19	-0.253	-0.0774	-0.26	-0.316	-0.0545	-0.0343	-0.128	0.158	0.0164	-0.171
					0.00811	0.00000033	0.0189	0.0246	0.000379	0.0583	0.0484	0.0102	0.0381	0.196	0.0819	0.599	0.0738	0.0286	0.712	0.816	0.383	0.281	0.911	0.243
**Lactate**						0.386	0.195	0.172	0.473	0.028	-0.042	-0.16	0.089	0.163	0.201	0.0542	-0.135	-0.206	0.0535	0.42	0.251	0.12	0.126	0.266
						0.00701	0.184	0.24	0.00075	0.849	0.776	0.276	0.546	0.266	0.17	0.713	0.359	0.16	0.717	0.0031	0.0847	0.414	0.391	0.0679
**N-acetylcysteine**							0.183	0.306	0.393	-0.286	-0.159	-0.128	-0.265	0.05	-0.0322	-0.223	-0.236	-0.229	-0.0956	0.0432	-0.144	0.38	0.117	-0.0817
							0.213	0.0343	0.0059	0.0488	0.278	0.385	0.0684	0.734	0.827	0.127	0.105	0.116	0.516	0.769	0.327	0.0079	0.425	0.579
**Fumarate**								0.175	0.312	-0.00038	-0.0944	-0.137	-0.217	-0.0841	-0.0856	0.0553	0.0972	-0.0235	-0.0111	0.136	0.135	0.0223	0.0769	-0.331
								0.234	0.0309	0.997	0.522	0.352	0.137	0.568	0.561	0.708	0.509	0.873	0.94	0.355	0.358	0.879	0.602	0.0216
**Choline**									0.574	-0.141	-0.249	-0.255	-0.0684	-0.16	-0.151	-0.144	-0.116	-0.111	0.411	0.128	0.0746	0.0331	0.0614	0.0706
									0.0000229	0.339	0.0874	0.0797	0.643	0.276	0.305	0.328	0.43	0.45	0.00391	0.386	0.613	0.822	0.677	0.632
**Erythritol**										-0.0506	-0.184	-0.195	-0.00109	-0.042	-0.111	-0.0704	-0.124	-0.123	0.39	0.449	0.336	-0.0169	0.155	0.0105
										0.732	0.209	0.184	0.994	0.776	0.449	0.633	0.4	0.405	0.00634	0.00146	0.0196	0.908	0.291	0.943
**Taurine**											0.775	0.69	0.665	0.658	0.733	0.674	0.62	0.727	0.306	0.381	0.431	-0.126	0.318	0.351
											0.0000002	0.0000002	4.37E-08	1.42E-07	0.0000002	0.0000002	0.00000262	0.0000002	0.0346	0.00771	0.00236	0.391	0.0278	0.0148
**Alanine**												0.86	0.664	0.682	0.827	0.598	0.577	0.758	0.0842	0.269	0.334	0.173	0.324	0.376
												0.0000002	4.78E-08	0.0000002	0.0000002	0.00000791	0.00002	0.0000002	0.568	0.0646	0.0207	0.238	0.0249	0.00863
**Glutamate**													0.571	0.651	0.71	0.531	0.548	0.74	0.0737	0.17	0.253	0.191	0.313	0.319
													0.0000263	3.08E-07	0.0000002	0.000118	0.0000631	0.0000002	0.617	0.247	0.0819	0.192	0.0303	0.0272
**TMAO**														0.583	0.585	0.533	0.241	0.409	0.293	0.37	0.315	0.0376	0.31	0.543
														0.0000159	0.0000145	0.000111	0.098	0.0041	0.0434	0.00991	0.0296	0.798	0.0319	0.0000768
**Isoleucine**															0.868	0.434	0.435	0.569	0.295	0.276	0.303	0.273	0.742	0.371
															0.0000002	0.00219	0.00215	0.0000288	0.042	0.0575	0.0367	0.0603	0.0000002	0.00964
**Valine**																0.484	0.514	0.686	0.157	0.283	0.342	0.209	0.566	0.38
																0.000534	0.000207	0.0000002	0.285	0.0513	0.0175	0.153	0.0000321	0.00799
**Glycine**																	0.469	0.55	0.015	0.366	0.355	0.0719	0.162	0.236
																	0.000832	0.0000593	0.918	0.0108	0.0135	0.626	0.269	0.105
**Carnitine**																		0.832	0.16	0.117	0.35	0.0618	0.171	-0.0417
																		0.0000002	0.276	0.426	0.0151	0.675	0.244	0.777
**Glutamine**																			0.201	0.262	0.395	0.049	0.261	0.116
																			0.17	0.0723	0.00566	0.739	0.0731	0.432
**Betaine**																				0.334	0.356	-0.0796	0.306	0.0804
																				0.0206	0.0131	0.589	0.0347	0.585
**Glucose**																					0.571	-0.0217	0.198	0.364
																					0.0000262	0.883	0.176	0.0112
**Guanidoacetate**																						-0.0893	0.249	0.0394
																						0.544	0.0882	0.789
**N-acetylglutamine**																							0.311	0.25
																							0.0319	0.0859
**Leucine**																								0.214
																								0.144
**Threonine**																								

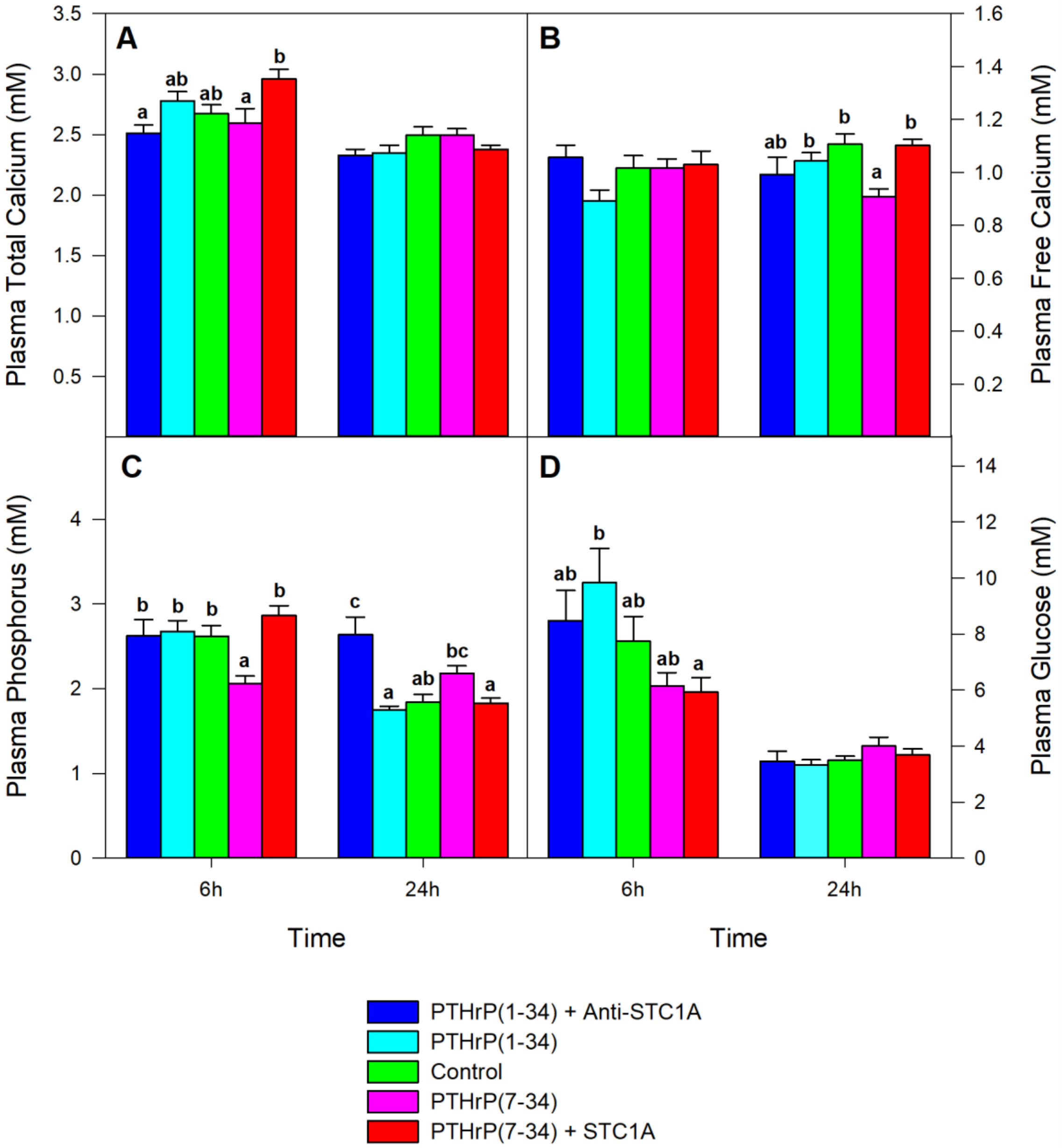

Supplementary Figure 1: Circulating plasma levels of (**A**) total calcium, (**B**) free calcium, (**C**) phosphorus and (**D**) glucose in the experimental groups (mM, mean ± SEM): control (green), PTHrP(1-34) (cyan), PTHrP(1-34)+Anti-STC1A (blue), PTHrP(7-34) (magenta) and PTHrP(7-34)+STC1A (red). To the left of the control (green) are the pro-PTHrP groups and to the right the pro-STC1 groups. Different letters denote significant differences between groups ($p < 0.05$, one-way ANOVA).

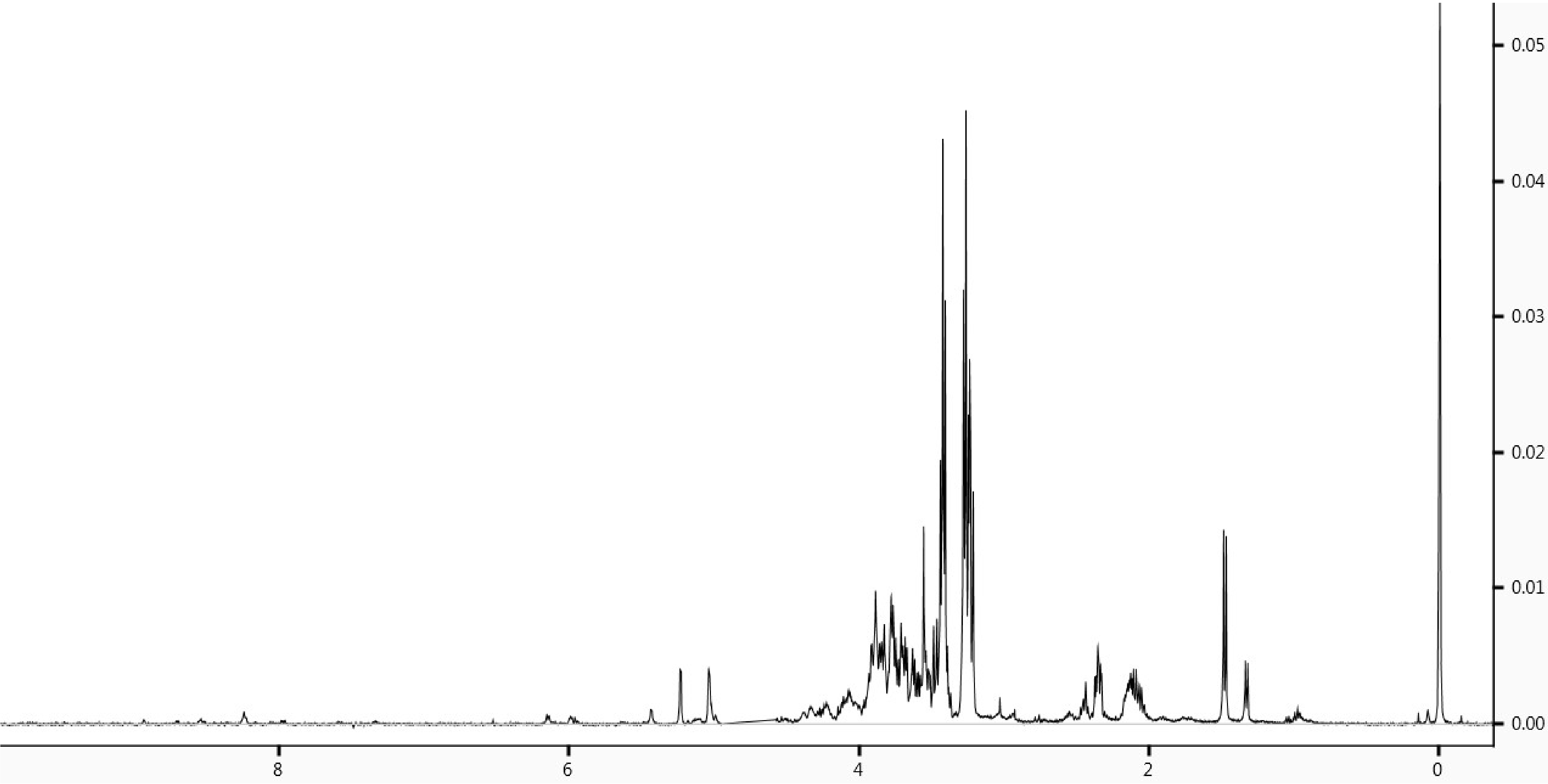

Supplementary Figure 2: Example of ^{1}H-NMR spectrum of a European sea bass liver extract.

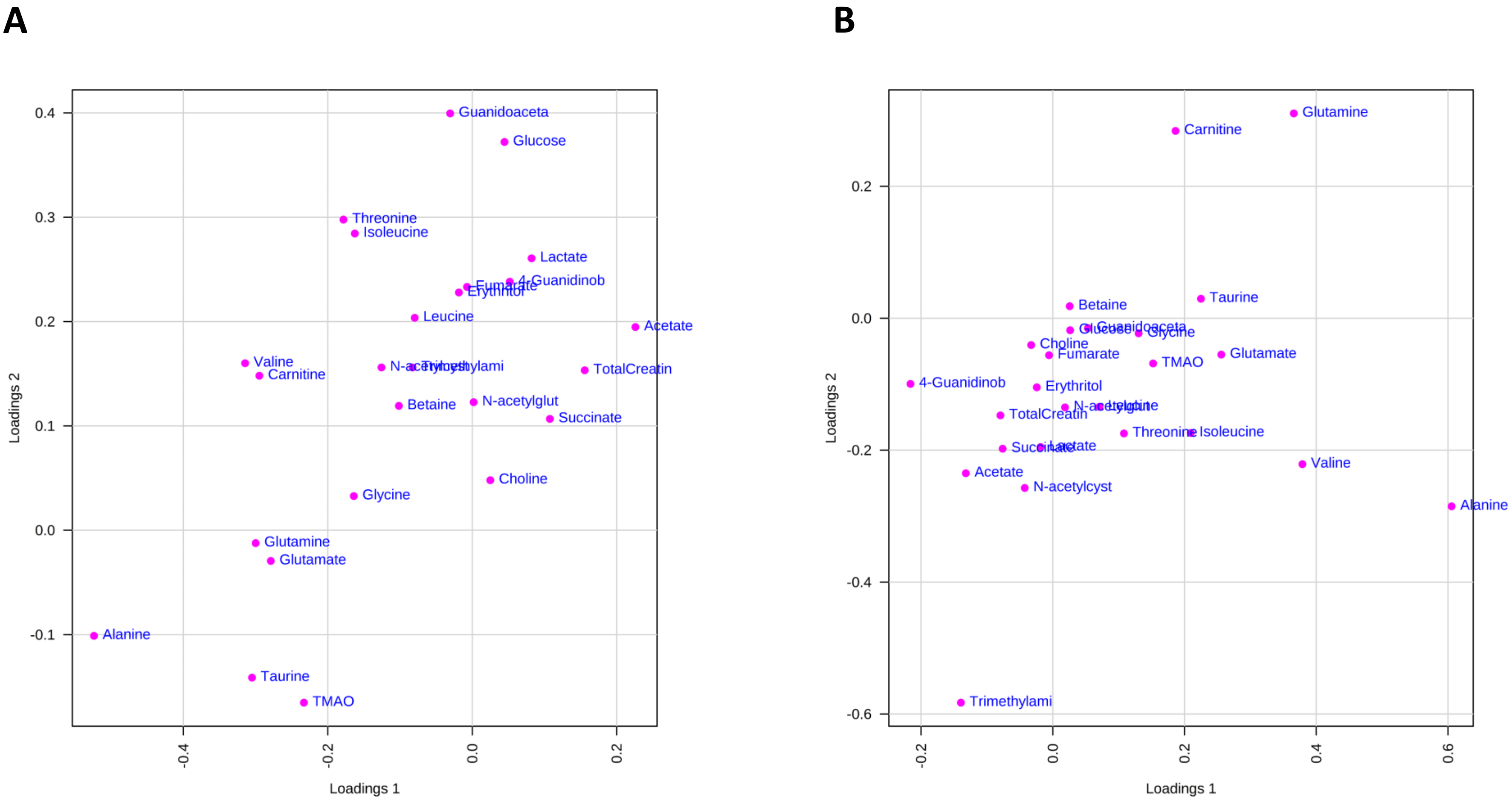

Supplementary Figure 3: PCA loading plot for the first two principal components at 6 h **(A)** and at 24 h **(B)** of hormone treatments. The circles indicate the most influential data points located on the outermost areas along the direction of separation as identified in the corresponding score plot.

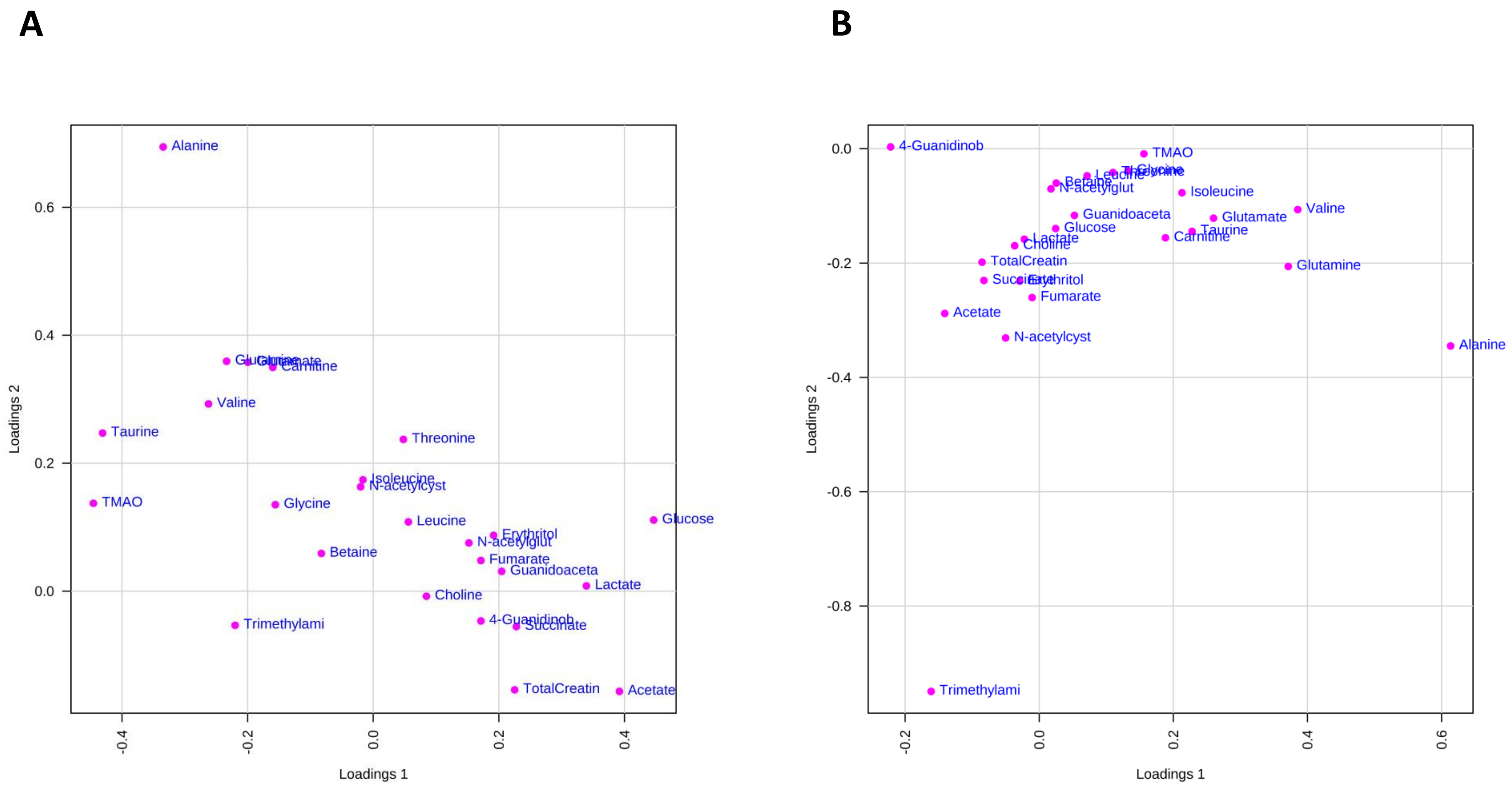

Supplemental Figure 4: PLS-DA loading plot between the first two components at 6 h **(A)** and at 24 h **(B)** of hormone treatments. The circles indicate the most influential data points located on the outermost areas along the direction of separation as identified in the corresponding score plot.
